# Whole‐genome sequencing based discovery of candidate genes and diagnostic markers for seed weight in groundnut

**DOI:** 10.1002/tpg2.20265

**Published:** 2022-12-07

**Authors:** Sunil S. Gangurde, Aamir W. Khan, Pasupuleti Janila, Murali T. Variath, Surendra S. Manohar, Prashant Singam, Annapurna Chitikineni, Rajeev K. Varshney, Manish K. Pandey

**Affiliations:** ^1^ Center of Excellence in Genomics & Systems Biology (CEGSB), International Crops Research Institute for the Semi‐Arid Tropics (ICRISAT) Hyderabad 502324 India; ^2^ Dep. of Genetics Osmania Univ. Hyderabad 500007 India; ^3^ State Agricultural Biotechnology Centre, Crop Research Innovation Centre, Food Futures Institute Murdoch Univ. Murdoch Western Australia 6150 Australia

## Abstract

Seed weight in groundnut (*Arachis hypogaea* L.) has direct impact on yield as well as market price because of preference for bold seeds by consumers and industry, thereby making seed‐size improvement as one of the most important objectives of groundnut breeding programs globally. Marker‐based early generation selection can accelerate the process of breeding for developing large‐seeded varieties. In this context, we deployed the quantitative trait locus‐sequencing (QTL‐seq) approach on a biparental mapping population (Chico × ICGV 02251) to identify candidate genes and develop markers for seed weight in groundnut. A total of 289.4–389.4 million reads sequencing data were generated from three libraries (ICGV 02251 and two extreme bulks) achieving 93.9–95.1% genome coverage and 8.34–9.29× average read depth. The analysis of sequencing data using QTL‐seq pipeline identified five genomic regions (three on chromosome B06 and one each on chromosomes B08 and B09) for seed weight. Detailed analysis of above associated genomic regions detected 182 single‐nucleotide polymorphisms (SNPs) in genic and intergenic regions, and 11 of these SNPs were nonsynonymous in the genomic regions of 10 candidate genes including *Ulp proteases* and *BIG SEED locus* genes. Kompetitive allele specific polymerase chain reaction (KASP) markers for 14 SNPs were developed, and four of these markers (snpAH0031, snpAH0033, snpAH0037, and snpAH0038) were successfully validated for deployment in breeding for large‐seeded groundnut varieties.

## Core ideas

- QTL‐seq analysis identified three genomic regions associated with groundnut seed weight were on chromosomes B06, B08, and B09.
- Eleven non‐synonymous SNPs identified in the regions of 10 important candidate genes associated with seed weight in groundnut.
- The *BIG SEEDs* locus associated with groundnut seed weight was identified in genomic region on chromosome B09.
- Four diagnostic KASP markers were developed and validated for deploying genomic‐assisted breeding to improve seed weight in groundnut.

**Abbreviations**
100SW: 100‐seed weight
HS: high‐seed weight
LS: low‐seed weight
MEGS: marker‐based early generation selection
PCR: polymerase chain reaction
QTL: quantitative trait locus
QTL‐seq: quantitative trait locus sequencing
RIL: recombinant inbred line
SNP: single‐nucleotide polymorphism

## 1 INTRODUCTION

Groundnut, or peanut (*Arachis hypogaea* L.), is an important oilseed and food crop cultivated in semi‐arid regions of the world. This crop is cultivated on over 31.6 million ha with an annual production of 53.6 Tg (http://www.fao.org/faostat/en). Groundnut is a nutritious legume crop that is known for its healthy oil, protein, and fiber content. Although the combination of big seed size and high oil content ensures higher pod yield and oil yield, the combination of big seed size and low oil content ensures industry and consumer satisfaction. Bold‐seeded groundnut is generally preferred in confections, being an important physical quality that attracts the immediate attention of consumers (Venuprasad et al., 2011). Groundnut with both big seed size and high oil content have great potential to benefit groundnut farmers, industry, and, most importantly, consumers.

The size of the pod and seed in groundnut are positively correlated, whereas the relationship between pod size and shelling percentage is not always positive, which limits success in developing varieties bearing large pods with high shelling percentage (de Godoy & Norden, 1981). Seed size has been one of the important domesticated traits in breeding groundnut varieties with different growth habits (Runner and Spanish). In general, Runner type varieties, having large seeds, are suitable for food purposes, whereas Spanish type, with small seed size, are mostly grown for oil extraction. Because of multiple preparations being available in the market for food usage, there is an increased demand of groundnut with more market‐preferred features such as big seed size and high oleic acid. This trend is growing more in Asia and Africa where the major proportion of produce is used for oil extraction. It is very crucial to study the genetic basis of regulation of seed weight in groundnut to discover candidate genes and to develop diagnostic markers that can be used in marker‐based early generation selection (MEGS) for improving the seed weight of elite groundnut cultivars.

Genomics‐assisted breeding has been playing an important role in groundnut, and there are many examples of successful efforts in developing improved varieties with disease resistant and high oleic acid content using diagnostic markers (see Pandey et al., 2020; Varshney et al., 2021). The availability of linked markers for seed weight will provide opportunity to perform MEGS in breeding bold‐seeded groundnut varieties. It is worth mentioning here that phenotypic selection for seed size is very difficult in groundnut, as the pods are inside the soil and the breeder needs to dig out pods in order to have an idea of pod and seed size. Under such scenario, the MEGS for seed size will provide great support to make decisions on other important traits in further generations. The previous genetic mapping efforts in groundnut for seed weight identified three significant quantitative trait loci (QTLs) located in a 2.7‐Mb region at the end of chromosome A05 (Luo et al., 2017) as well as pod weight on chromosome A05 (Luo et al., 2018) and a major QTL on chromosome A05 for seed number per pod (Chen et al., 2019a). The meta‐QTL analysis using consensus map narrowed down the genomic region for seed weight from 2.7 to 0.7 cM on chromosome A05(Lu et al., 2018). Further, a recent nested‐association mapping approach in groundnut identified associated genomic loci on chromosomes A05, A06, A08, A09, B05, B06, B08, and B09 for seed weight (Gangurde et al., 2020). None of these studies, however, reported development of diagnostic markers for seed size to accelerate breeding for large‐seeded groundnut varieties.

**Core ideas**

- QTL‐seq analysis identified three genomic regions associated with groundnut seed weight were on chromosomes B06, B08, and B09.
- Eleven non‐synonymous SNPs identified in the regions of 10 important candidate genes associated with seed weight in groundnut.
- The *BIG SEEDs* locus associated with groundnut seed weight was identified in genomic region on chromosome B09.
- Four diagnostic KASP markers were developed and validated for deploying genomic‐assisted breeding to improve seed weight in groundnut.

Availability of reference genomes coupled with the next‐generation sequencing technologies have accelerated candidate gene discovery and marker development followed by their deployment in breeding including in groundnut (see Pandey et al., 2016; Varshney et al., 2019; Bertioli et al., 2019; Zhuang et al., 2019). Among these approaches, a next‐generation sequencing‐based bulk segregant analysis approach, also known as QTL sequencing (QTL‐seq), has been widely used for discovery of genomic regions and candidate genes as well as diagnostic markers. This approach provided successful results for resistance to late leaf spot and rust (Pandey et al., 2017; Clevenger et al., 2018) and bacterial wilt (Luo et al., 2019a), shelling percentage (Luo et al., 2019b), testa color (Zhao et al., 2020), and fresh‐seed dormancy (Kumar et al., 2020) in groundnut. The QTL‐seq approach uses the advantage of sequencing and generates thousands of genome‐wide single‐nucleotide polymorphism (SNP) variants for bulks by using reference‐guided assembly of either parent of a recombinant inbred line (RIL) population (Takagi et al., 2013). In the present study, we used QTL‐seq approach to study the molecular basis of seed weight in groundnut by using a RIL population and identified associated genomic regions and candidate genes for seed weight in addition to development and validation of diagnostic markers for MEGS.

## 2 MATERIALS AND METHODS

### 2.1 Plant materials and construction of bulks with extreme phenotypes

**FIGURE 1:**
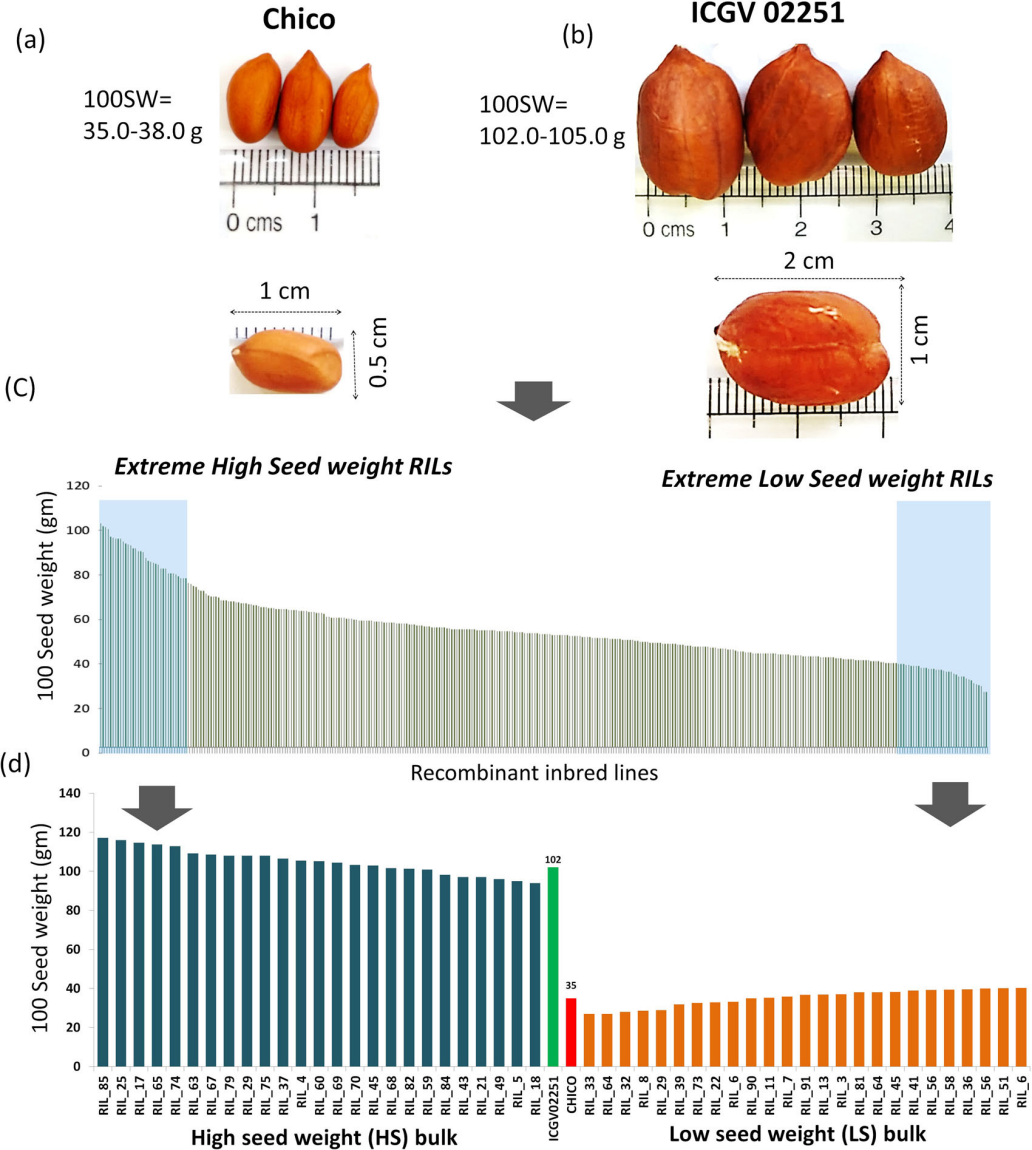
Construction of bulks with extreme phenotypes for 100‐seed weight (100SW). (a) Chico, low‐100SW parent for 100SW. (b) ICGV 02251, high 100SW parent for 100SW. (c) Frequency distribution for mean 100SW values of recombinant inbred line (RIL) population. These mean values were calculated based on phenotyping data generated in 2014 at ICRISAT. (d) Phenotypic variability among the RILs selected for development of extreme bulks for 100SW. Based on the phenotyping of RIL population, 25 high 100SW RILs and 25 low 100SW RILs were used to constitute low‐seed weight and high‐seed weight bulks

A RIL population (Chico × ICGV 02251) comprising of 385 lines (RILs) was developed by crossing groundnut cultivars Chico, a small‐seeded cultivar, and ICGV 02251 a large‐seeded genotype (Figure 1a,b). Extensive phenotyping data for 100‐seed weight (100SW) was generated on a RIL population in the F_9_ generation and was phenotyped at ICRISAT, Hyderabad, India, during three seasons (rainy 2014, postrainy 2014–2015, and rainy 2019). The extreme pools were prepared for 100SW based on phenotyping data obtained for the year postrainy 2014–2015. For developing the extreme bulks for trait 100SW, 25 RILs with high mean seed weight and 25 RILs with low mean seed weight were selected. The equimolar concentration of DNA from 25 RILs with high mean seed weight were pooled together to construct the high‐seed‐weight (HS) bulk; similarly, DNA from RILs with low mean seed weight was pooled together to construct the low‐seed‐weight (LS) bulk. Thus, two extreme bulks were prepared for sequencing. High‐seed‐weight parent ICGV 02251 was used as a reference parent to calculate SNP indices for LS and HS bulks (Figure 1d).

### 2.2 Construction of libraries and sequencing for bulks and parent

The whole‐genome resequencing data were generated for three samples, namely ICGV 02251 (bold‐seeded parent), HS bulk, and LS bulk, and were prepared and used for sequencing on Illumina HiSeq 2500 at the Center of Excellence in Genomics and Systems Biology (CEGSB; cegsb.icrisat.org), ICRISAT, Hyderabad, as described in Pandey et al. (2017). Sequencing data generated have been deposited at National Center for Biotechnology Information (NCBI) Sequence Read Archive database with BioProject ID PRJNA752462. In brief, a single Illumina library for each sample was made using TruSeq DNA Sample Prep kit. Two micrograms of DNA from each of these three samples was first sheared using diagenode Bioruptor NGS and then was subjected to end repairing and adapter ligation. Two percent agarose gel was used for selection of libraries on the basis of size. Further, the libraries were enriched using adaptor‐compatible polymerase chain reaction (PCR) primers. A chip assay was performed for size distribution of amplified DNA libraries and was checked on an Agilent Technologies 2100 Bioanalyzer. The DNA libraries were sequenced on Illumina HiSeq 2500 (Illumina Inc.) to generate 250 bp paired‐end reads.

### 2.3 Development of reference guided assembly and QTL‐seq analysis

The quality assessment and data estimation before and after quality control was performed using Raspberry of NGS‐QC box (Katta et al., 2015). The cleaned reads of parent ICGV 02251 were first aligned to the combined assembly of groundnut progenitors (A‐ and B‐diploid subgenomes) (Bertioli et al., 2016) using BWA aligner (Li and Durbin, 2009). Postprocessing and filtering of the alignment files was performed using Coval (Kosugi et al., 2013). The variants called after aligning the reads were then used to develop reference‐based assembly of the parent (ICGV 02251) by substituting the bases with high confidence variants in the genome. The reads from HS and LS bulks for 100SW were then aligned to ICGV 02251 assembly and variants were called for HS and LS bulks by using the QTL‐seq pipeline developed by the Iwate Biotechnology Research Center, Japan (Takagi et al., 2013).

### 2.4 Calculation of SNP index to identify associated SNPs

The SNP index for each SNP at its respective genomic position was calculated for HS and LS bulks as per the formula given in Abe et al. (2012), Takagi et al. (2013), Pandey et al. (2017), and Luo et al. (2019a, 2019b). The SNPs at positions with high read depth of <10 in both the bulks and SNP index of <0.3 in either of the bulks were filtered out for calculation of ΔSNP index. Only SNP positions with ΔSNP index = −1 were considered as the causal SNPs and associated with 100SW. The possible effects of the identified SNPs were inferred using SnpEff v3.0 open‐source program (Cingolani et al., 2012). Further, the annotations of SNPs associated with 100SW were searched on diploid and tetraploid genomes (www.peanutbase.org), and corresponding candidate genes affected by the causal SNPs were discovered.

### 2.5 KASP marker development and validation

The SNPs with ΔSNP index = −1 affecting the function of important candidate genes were used for marker development. The SNPs were selected from the regions near to candidate genes in the four genomic regions on three different chromosomes. The developed Kompetitive allele specific PCR (KASP) markers were validated in contrasting germplasm lines from ICRISAT. In this context, the SNPs were converted into KASP markers by using 50‐bp upstream and 50‐bp downstream sequences for the development of user‐friendly and cost‐effective markers (He et al., 2014). While designing the KASP assays for each SNP marker, two allele‐specific forward primers and one common reverse primer was designed (Intertek Pvt. Ltd.). All the KASP primers used in this study are listed in Supplemental Table S1. The developed KASP markers were validated on a validation panel of 39 genotypes comprised of 18 bold‐seeded, 16 small‐seeded, and five F_1_ generation lines derived from small‐ and bold‐seeded genotypes (Supplemental Table S2).

## 3 RESULTS

### 3.1 Phenotyping and construction of extreme HS and LS bulks

A RIL population (Chico × ICGV 02251) comprising 350 RILs was phenotyped for 100SW. The 100SW of large‐seeded parent ICGV 02251 was 98.2 ± 0.62 g, whereas that of small‐seeded Chico was 35.3 ± 0.42 g (Figure 1a,b). The population showed high phenotypic variability for 100SW (Supplemental Table S3), ranging from 28.7 to 117.2 g with normal distribution and partial transgressive segregation over both parents (Figure 1c). Based on the phenotyping data, 25 extreme RILs with high seed weight (90.3–117.2 g) and 25 RILs with low seed weight (28.7–41.5 g) were selected to construct HS and LS bulks (Figure 1d), respectively. The average 100SW of HS bulk was 102.1 g, while that of LS bulk was 37.6 g.

### 3.2 Whole‐genome sequencing of bulks and SNP discovery

Both bulks (HS and LS), along with bold‐seeded parent ICGV 02251, were used for construction of Illumina sequencing libraries and sequenced using Illumina HiSeq 2500. A total of 289.4 million reads (36.2 Gb) were generated for bold‐seeded parent ICGV 02251, 329.4 million reads (41.2 Gb) for HS bulk and 319.2 million reads (39.9 Gb) for the LS bulk (Table 1). After quality control analysis of high‐quality reads, a total of 210.6 million reads for ICGV 02251, 233.0 million reads for HS bulk, and 222.9 million reads for LS bulk, they were aligned on diploid reference genomes (https://peanutbase.org/peanut_genome) (Supplemental Table S4). A reference‐guided assembly for parent ICGV 02251 was developed, which achieved 93.9% alignment and 85.9% genome coverage on reference genome with 8.34× of average read depth. Quality reads of HS bulk were mapped on a reference‐guided assembly and resulted in 95.0% alignment and 85.9% genome coverage with average depth of 9.27×. Similarly, the mapping of LS bulk achieved 95.1% alignment and 86.2% genome coverage with average depth of 9.29× (Table 1; Supplemental Table S4). The comprehensive sequence analysis for detection of sequence variants between HS and LS bulks identified a total of 2,22,423 genome‐wide SNPs of which 106,850 SNPs (48% of total SNPs) were homozygous SNPs. The SNP index was calculated for all the SNPs detected between the two bulks and plotted against SNP position in the genome and represented graphically. Further, these SNP indices from the HS and LS bulks were used for calculation of the ΔSNP index (Supplemental Table S5, Supplemental Figures S1–S3).

**TABLE 1:** Summary of Illumina sequencing of parental line and bulks for 100‐seed weight in groundnut

**Sample ID**	**Total reads (raw)**	**Total reads after quality control**	**Data generated**	**Genome coverage**	**Average mapping depth (×)**	**Alignment percentage**
			Gb	%		%
ICGV 02251	289,451,760	201,662,379	36.18	85.89	8.34×	93.93
HS bulk	329,428,944	233,019,623	41.18	85.90	9.27×	95.00
LS bulk	319,222,984	222,958,479	39.91	86.16	9.29×	95.12

*Note*. HS bulk, high‐seed‐weight bulk; LS bulk, low‐seed‐weight bulk.

### 3.3 Candidate genomic regions for seed weight

**FIGURE 2:**
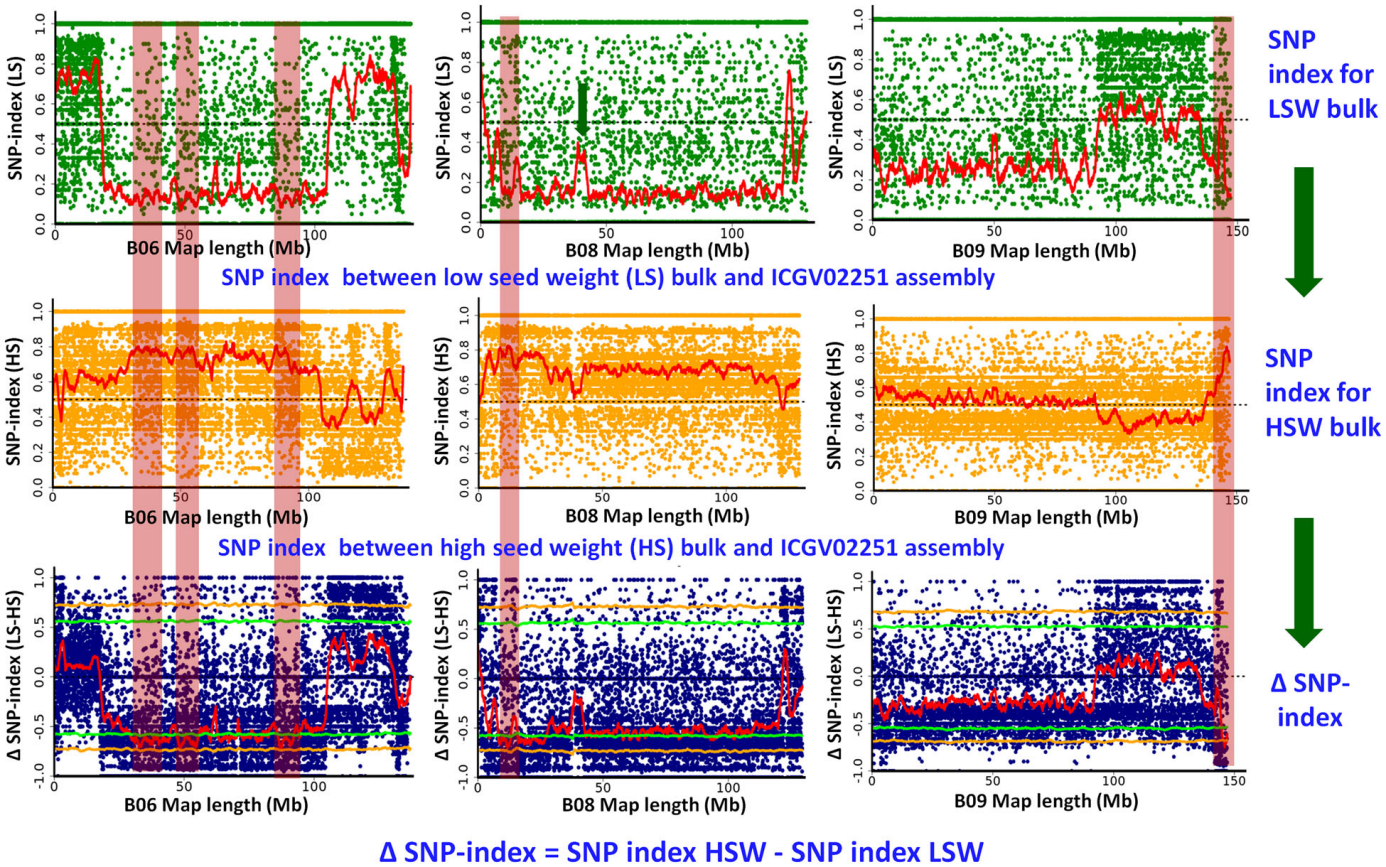
Quantitative trail locus‐sequencing approach for mapping genomic regions controlling seed weight using the ICGV 02251 as reference parent. Single‐nucleotide polymorphism (SNP) index plot between low bulk and ICGV 02251 assembly (green color), high bulk and ICGV02251 assembly (yellow color), and ΔSNP index plot (blue color) on chromosomes B06, B08, and B09 with statistical confidence interval under the null hypothesis of no QTL (orange, *P* < .01 and green *P* < .05). The associated genomic region is shaded in pink color on all three chromosomes

The SNP index was calculated for HS and LS bulks to discover the genomic regions responsible for 100SW. The deviation of SNP index from 0.5 to 0 indicates the proportion of more alleles contributing from one parent, and 0.5 to 1.0 indicates the proportion of alleles contributing more from another parent. Three significant genomic regions linked to seed weight were identified on chromosomes B06 and one genomic region each on chromosome B08 and B09 using the criteria of a sliding window of 2.0‐Mb intervals with 50‐kb region increasing on physical map and ΔSNP index with a statistical confidence of *P* < .01 between the HS and LS bulks (Supplemental Figures S1–S3). Furthermore, after analyzing the sequences of LS and HS bulks with ICGV 02251 assembly as a reference, 104 SNPs in three genomic regions were identified on chromosome B06. Of these regions, the first genomic region of 8.9 Mb (31.57–40.52 Mb) harbored 55 SNPs, the second region of 1.1 Mb (53.56–54.66 Mb) had 21 SNPs, while the third genomic region of 1.64 Mb (81.17 –82.81 Mb) contained 28 SNPs. Similarly, one 2.64 Mb (10.95–13.59 Mb) region containing 48 SNPs was identified on chromosome B08, while a 930‐Kb (145,797,595–146,728,008 bp) region harboring 30 nonsynonymous SNPs was identified on chromosome B09. The negative ΔSNP index indicates presence of alleles in the bulks from parental genome of ICGV 02251 (Figure 2). We also confirmed the presence of the associated genomic regions identified in the present study on chromosome B06 (logarithm of the odds = 4.9; percentage variance explained = 22.3%) and B09 (logarithm of the odds = 6.6; percentage variance explained = 10.6%) by performing genetic mapping and QTL discovery using the same RIL population (Supplemental Table S6).

### 3.4 Discovery of highly significant SNPs and putative candidate genes for seed weight

**FIGURE 3:**
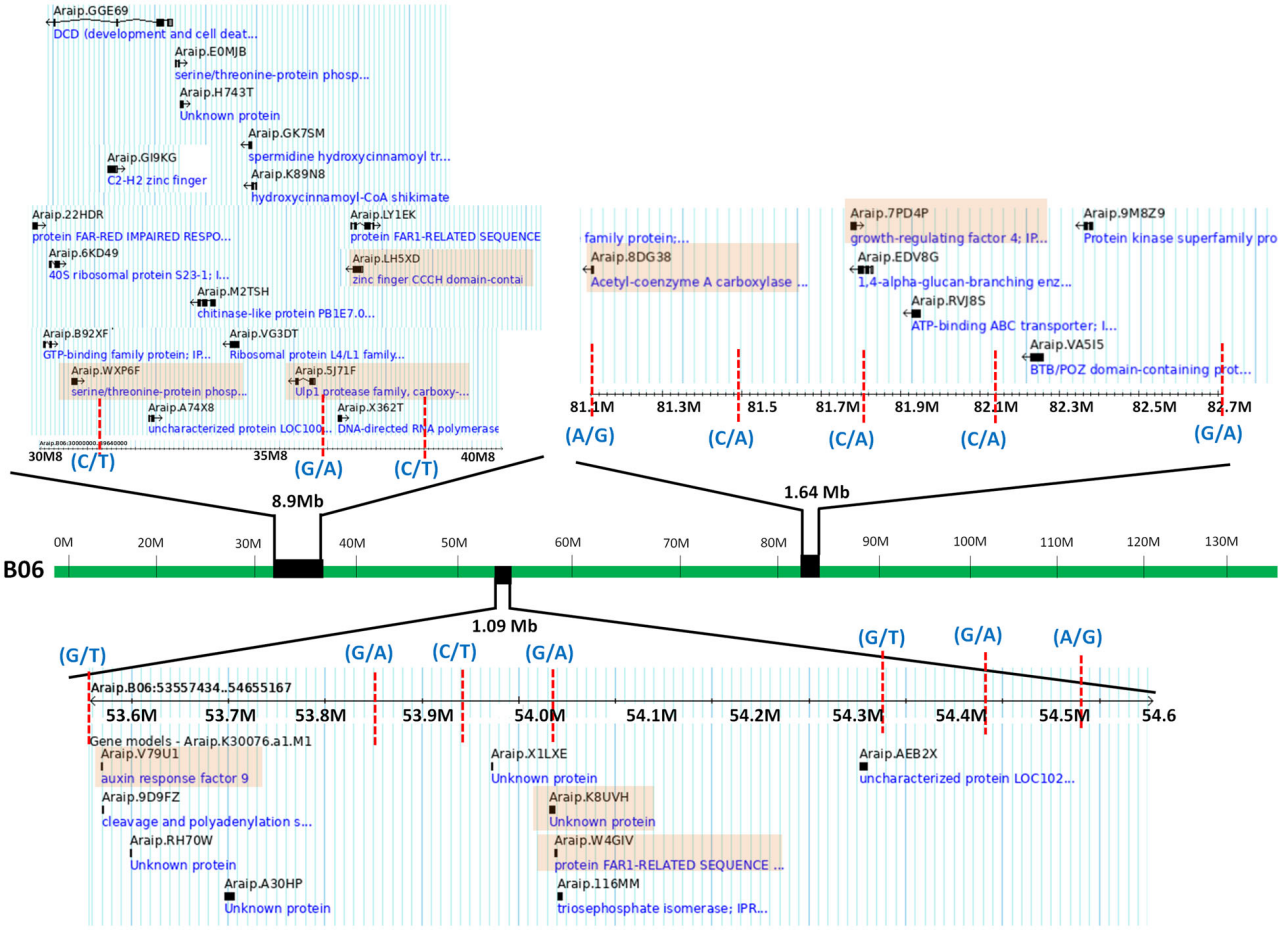
Important genes identified in the genomic region on the chromosome B06. Magnified view of regions from peanut base harboring nonsynonymous single‐nucleotide polymorphisms (SNPs) in various genic and intergenic regions. Nonsynonymous SNPs are indicated in vertical red dotted lines with identified SNP change. Seed and pod development related genes such as *Ulp1 proteases* (a SUMO conjugate), *auxin responsive factor*, *acetyl coenzyme A*, *growth regulating factor 4*, and some *unknown proteins* were located in the three genomic regions on chromosome B06

The SNP index analysis using original genome assembly and ICGV 02251 assembly reference with combination of HS and LS bulks detected three promising genomic regions on chromosome B06 associated with seed weight. These genomic regions harbored 104 effective SNPs with read depth ≥ 7; the SNP index significantly deviated from 0.5 and ΔSNP index −1, which is higher than the statistical confidence of *P* < .01 down to the line of 99% confidence interval at upper and lower side (Figure 2; Supplemental Table S7). The candidate genes identified based on diploid reference genomes were also found in corresponding genomic regions of the tetraploid reference genome (Supplemental Table S8). Of the 104 SNPs, 102 SNPs were located in intergenic region and two SNPs in the genic region (one each in intronic and exonic region). The SNP B06_54033720 was located in intronic region of a candidate gene *Ulp1 protease*, while the nonsynonymous SNP (B06_32002355) was located in the coding region of *unknown protein* (*Araip.K8UVH*) (Figure 3) according to the annotations of diploid as well as tetraploid genomes (www.peanutbase.com).

**FIGURE 4:**
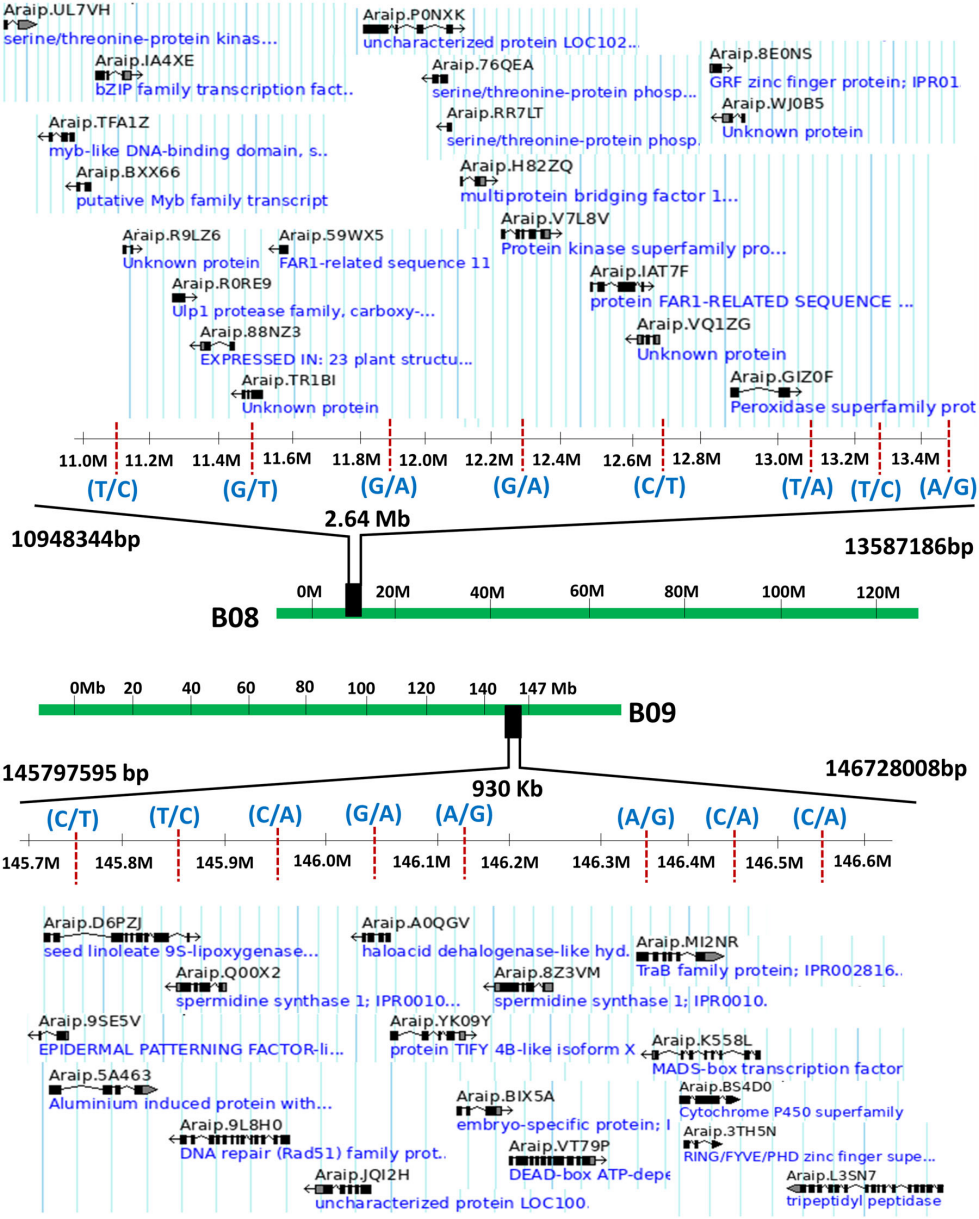
Important genes identified in the genomic region on the chromosomes B08 and B09. Magnified view from peanut base of a region harboring nonsynonymous single‐nucleotide polymorphisms (SNPs) in various intergenic regions. Nonsynonymous SNPs are indicated in vertical red dotted lines with identified SNP change. Seed and pod development related genes such as *serine/threonine protein phosphatases* and *serine/threonine protein kinases* were identified on chromosome B08 in the 2.64‐Mb genomic region. Whereas, spermidine synthase and seed linoleate *MADS box* and *DEAD box* genes were located in the 930‐Kb region on chromosome B09

The genomic region of 2.6 Mb on chromosome B08 harbored 48 SNPs, and five of these SNPs were located in intronic regions of four different candidate genes. The first SNP (B08_10998096) was located in the genic region of candidate gene *Serine/threonine‐protein kinase* (*Araip.UL7VH*), while two SNPs, namely B08_11007755 and B08_11007758, were present in the single genic region *basic leucine zipper domain* (*Araip.IA4XE*). The fourth SNP was located at B08_11594032 in the candidate gene *uncharacterized protein* (*Araip.P0NXK*), while the fifth SNP, B08_11696813, was in genic region of a candidate gene *Araip.WJ0B5* on chromosome B08 coding for unknown protein (Figure 4).

**FIGURE 5:**
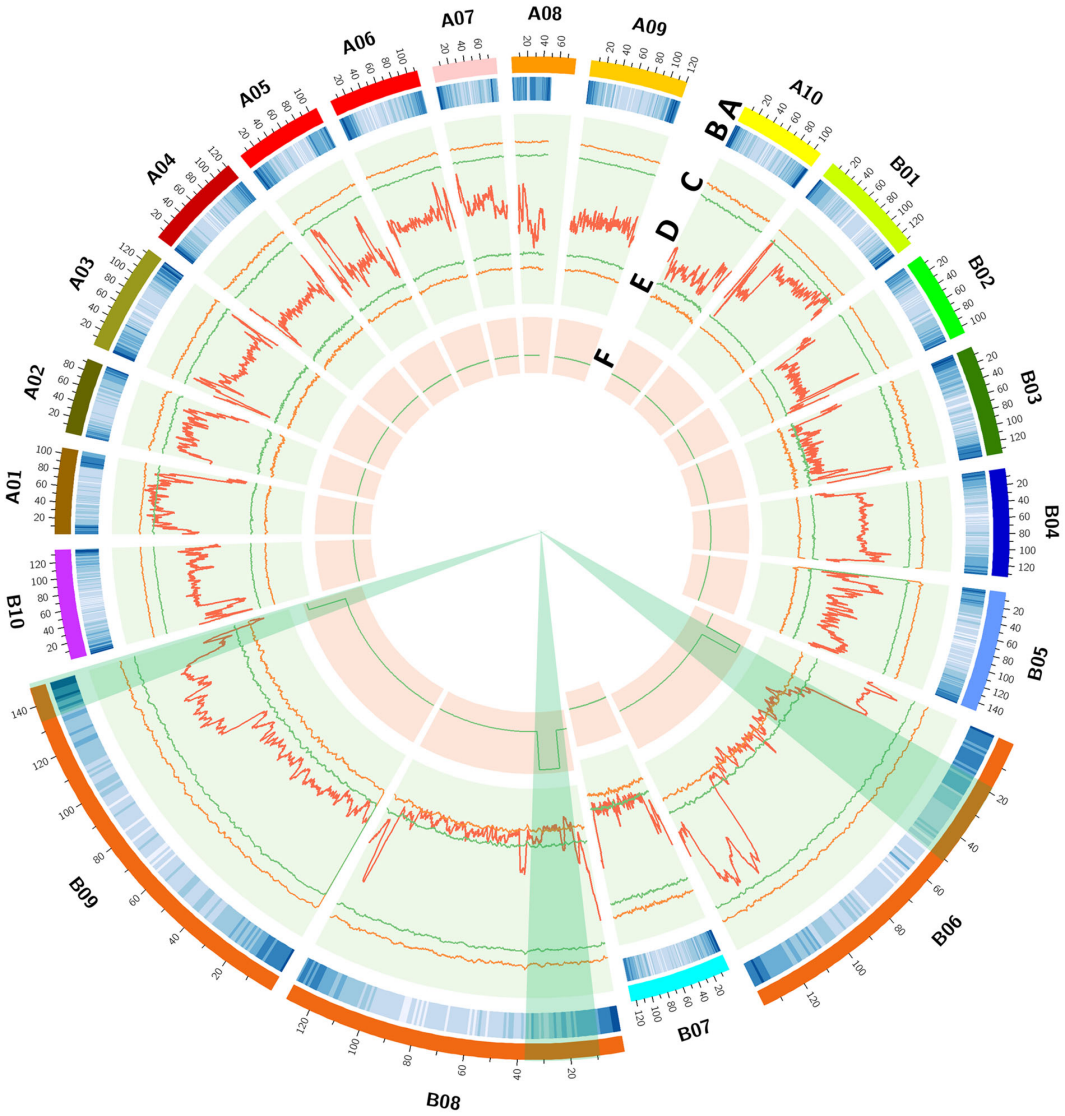
Circos plot represents genome‐wide ΔSNP index on 20 chromosomes of groundnut colocalized in previously reported quantitative trait loci (QTL) from traditional genetic mapping. In the illustration, the tracks from outside to inside indicate (a) chromosomes of reference genome *Arachis duranensis* and *A. ipaensis*; (b) genome‐wide density of annotated genes on groundnut genomes; the density of genes is higher in telomere than centromeric region of each chromosome; (c) upper probability values at 99% confidence and upper probability values at 95% confidence; (d) ΔSNP index plot using the ICGV 02251 assembly as reference; (e) lower probability values at 95% confidence and lower probability values at 99% confidence; and (f) physical position of earlier mapped QTL for groundnut seed weight through nested association mapping based genome‐wide association study and genetic mapping (Gangurde et al., 2020)

A region of 930 Kb detected on chromosome B09 housed 30 SNPs, and six of these SNPs were found affecting the function of six candidate genes. The first SNP at B09_145870014 affected the function of a candidate gene *methyltransferase* (*Araip.0U7×2*), while second SNP located at B09_145954466 affected candidate gene *EPIDERMAL PATTERNING FACTOR* (*Araip.9SE5V*). A third SNP at position B09_146042834 affected candidate gene *Aluminum induced protein* with *YGL* and *LRDR* (*Araip.5A463*), while a fourth SNP (B09_146042834) affected the candidate gene *MADS‐box transcription factor* (*Araip.K558L*). A fifth SNP (B09_146448322) affected the function of *TraB family protein* (*Araip.MI2NR*), while a sixth SNP (B09_146719055) affected the candidate gene *tripeptidyl peptidase* (*Araip.L3SN7*) (Figures 4 and 5).

**FIGURE 6:**
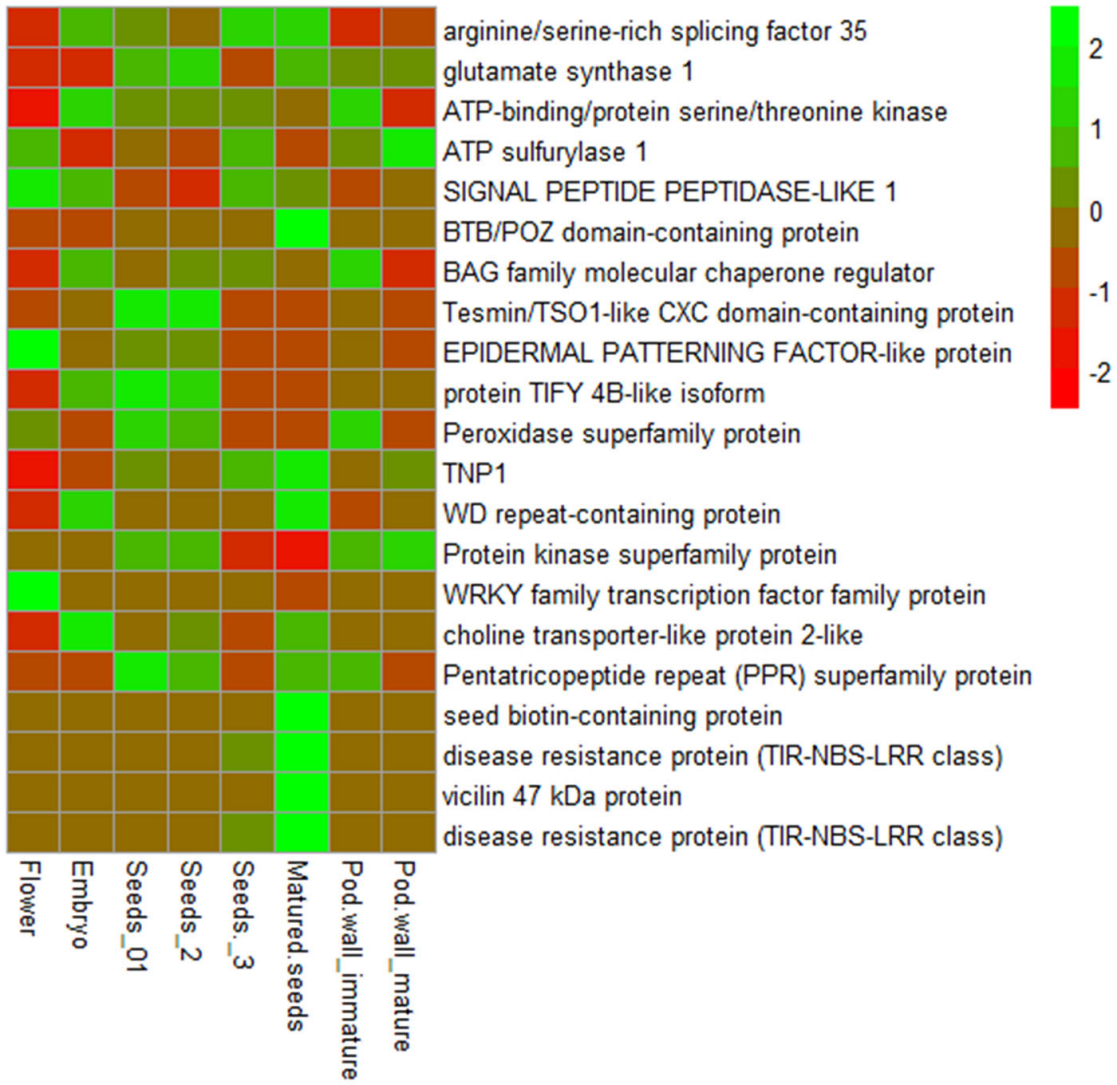
Expression of candidate genes especially expressed in flower, embryo, seed, and pod developmental stages based on gene expression atlas. Twenty‐one genes were expressed especially in seed, pod, embryo, and flower. The gene expression data was extracted from groundnut subspecies *fastigiata* gene expression (AhGEA) atlas (Sinha et al., 2020)

The expression of genes identified in the present study was studied using the recently developed groundnut gene expression atlas for the subspecies *fastigiata* (Sinha et al., 2020) and *hypogaea* (Clevenger et al., 2016) (Supplemental Tables S8 and S9). To check the specificity of gene expression in seed, we checked the expression levels of 49 candidate genes identified from three associated genomic regions located on chromosomes B06, B08, and B09 in the gene expression atlas. Of these 49 genes, 21 genes were highly expressed in flower, pod, and seed tissues. Three genes, namely *SIGNAL PEPTIDE PETIDASE*, *EPIDERMAL PATTERNING FACTOR*, and *WRKY* transcription factor, showed high expression in flower tissue. Four genes, including seed biotin containing protein, two isoforms of disease resistance *NBS‐LRR proteins*, and *vicilin 47‐kD protein TNP1*, were highly expressed in matured seed stage. Genes related to *TIFY family proteins*, *peroxidase super family proteins*, *pentatricopeptide repeats*, *glutamate synthase 1*, and *proteins kinases* showed high expression in developing seed. *Choline transporters* and *serine threonine ATP kinases* were highly expressed in embryo, while *ATP sulfurylase 1* was expressed in the matured pod wall (Figure 6).

### 3.5 Development and validation of KASP markers for seed weight

**FIGURE 7:**
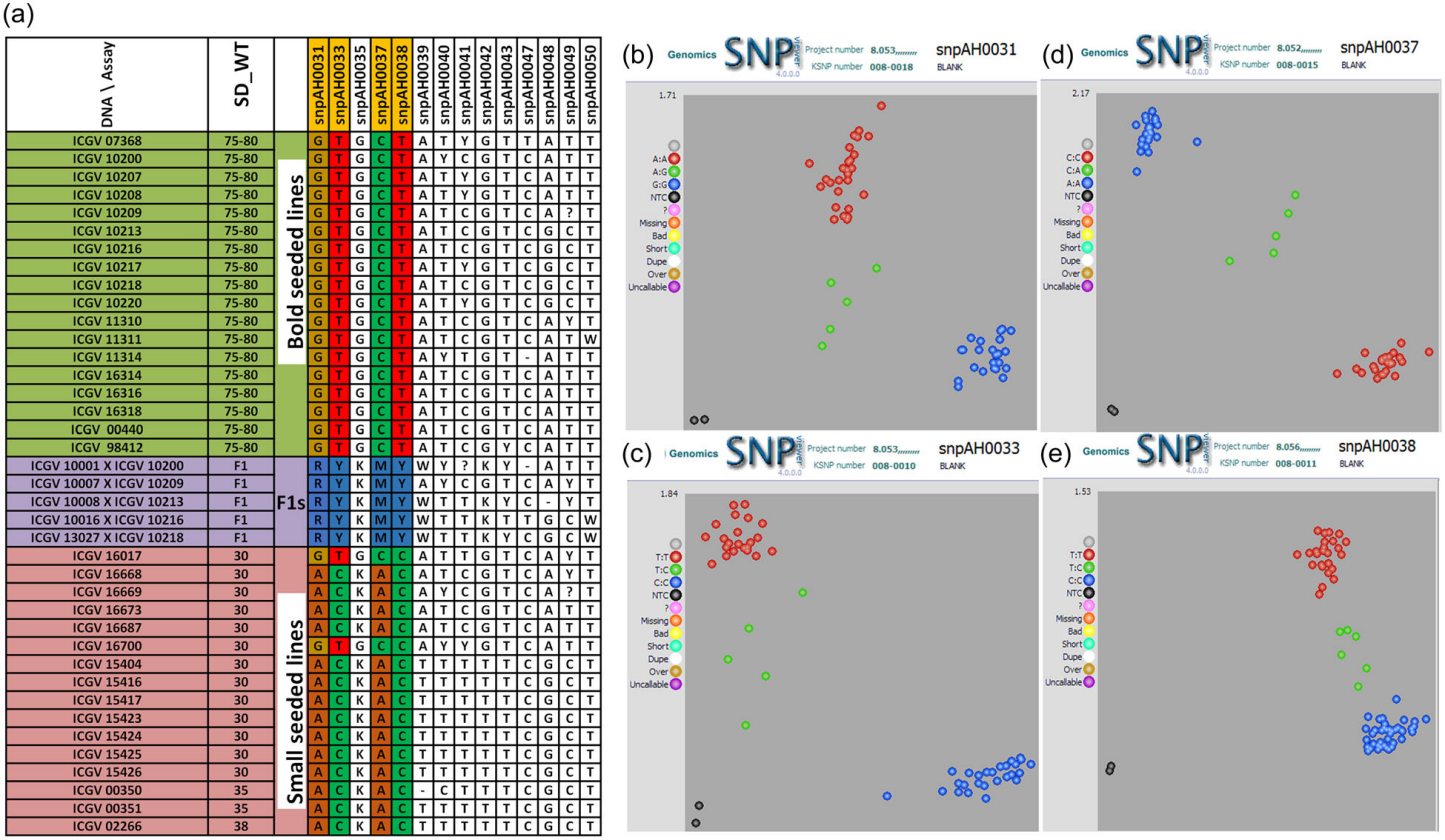
Development and validation of Kompetitive allele specific polymerase chain reaction (KASP) markers from potential candidate genes identified for seed weight. (a) Validation panel includes 18 bold‐seeded groundnut varieties (75–80 g 100‐seed weight), 16 small‐seeded groundnut varieties (25–30 g 100‐seed weight), and five F_1_ hybrids derived from different combinations of bold‐ and small‐seeded groundnut varieties. Four single‐nucleotide polymorphisms (SNPs) show clear homozygous clusters and one heterozygous cluster for heterozygotes for (b) snpAH0031 for gene *Ulp1 protease* (cysteine‐type peptidase activity) (*Araip.5J71F*), (c) snpAH0033 (intergenic SNP between *Araip.A3T8C* and *Araip.A5MIH*) for nearest gene ATP binding; *nucleoside‐triphosphatase*, (d) snpAH0037 (intergenic SNP between *Araip.VA5I5* and *Araip.9M8Z9*) for nearest gene protein kinase superfamily protein, (e) snpAH0038 (intergenic SNP between *Araip.9M8Z9* and *Araip.FJY6W*) for nearest gene protein kinase superfamily protein

Kompetitive allele specific PCR markers epitomized cost‐effective and less laborious assays of genotyping in order to perform early generation selection in breeding lines for indirect selection of a particular phenotype (Bohar et al., 2020). To develop and validate diagnostic markers for seed weight, 14 SNPs (five SNPs on B06, five SNPs on B08, and four SNPs on B09) were targeted for development of KASP markers. Primers were successfully developed for 14 SNPs and validated on a validation panel with contrasting seed weight (Supplemental Table S1). The validation panel was comprised of bold‐seeded lines with 100SW ranging from 75 to 80 g and small‐seeded lines with 100SW of 25–30 g. Five F_1_ populations derived from different crosses were also included in the panel to study the robustness of markers to detect heterozygosity (Supplemental Table S2). Of the 14 KASP markers validated, four KASPs (snpAH0031, snpAH0033, snpAH0037, and snpAH0038) showed expected polymorphism in the germplasm (Table 3). Interestingly, all these four polymorphic KASP markers differentiating genotypes with small‐ and bold‐seeded genotypes belonged to genomic region detected on chromosome B06 (Figure 7). The developed KASP markers were highly polymorphic between bold‐ and small‐seeded groundnut genotypes and can be used as potential diagnostic markers for selecting bold‐seeded segregating breeding material at the very early stage of varietal development process. In addition, the KASPs snpAH0039, snpAH0041, and snpAH0042 have moderately distinguished the genotypes on validation panel and showed specificity to certain genetic backgrounds to be used as diagnostic markers for seed weight. All validated KASP markers successfully confirmed F_1_ population lines derived from crosses of various small‐ and bold‐seeded lines.

## 4 DISCUSSION

Next‐generation sequencing technologies have revolutionized trait dissection, candidate gene discovery and have helped in developing cost‐effective genomic tools. Groundnut has entered in postgenome sequencing era with huge genomic resources (see Pandey et al., 2012, 2020). Availability of a high‐quality reference genome for both the progenitor diploid species (*A. duranensis* and *A. ipaensis*) (Bertioli et al., 2016; Chen et al., 2016) and subspecies of cultivated tetraploid groundnut (*A. hypogaea* subspp. *hypogaea* by Bertioli et al., 2019 and *A. hypogaea* subspp. *fastigiata* by Chen et al., 2019b and Zhuang et al., 2019). These genomic resources provided opportunity for developing genotyping assays (see Pandey et al., 2012; Varshney et al., 2013) in addition to sequencing‐based gene discovery (Pandey et al., 2016; Varshney et al., 2019). In addition to high‐quality reference genomes, global gene expression atlas has also become available for both the subspecies namely subspp. *fastigiata* (Sinha et al., 2020) and subspp. *hypogaea* (Clevenger et al., 2016).

Sequencing‐based trait mapping approaches, such as QTL‐seq, provides several advantages over traditional genetic mapping approaches as it leads to candidate gene discovery as well as diagnostic marker development. Following the principles of bulked segregant analysis (Michelmore et al., 1991), whole genome sequencing of pooled samples of extreme phenotypes in the QTL‐seq approach have been deployed earlier in groundnut, which facilitated faster discovery of candidate genes and markers for several important traits in groundnut including resistance to foliar fungal disease (Pandey et al., 2017; Clevenger et al., 2018) and bacterial wilt (Luo et al., 2019a), shelling percentage (Luo et al., 2019b), fresh seed dormancy (Kumar et al., 2020), and purple testa color (Zhao et al., 2020). Taking benefit of these developments and availability of reference genome sequences, we successfully used the QTL‐seq approach and identified genomic regions, candidate genes, and, more importantly, developed four diagnostic KASP markers associated with groundnut seed weight.

For performing the QTL‐seq experiment, we obtained 8.34–9.29× average read depth of sequencing data realizing the large genome size (2.7 GB) of cultivated tetraploid groundnut. Previous QTL‐seq experiments in groundnut used 10.3–11.6× average read depth for resistance to rust and late leaf spot (Pandey et al., 2017), ∼6.5× average depth for fresh seed dormancy (Kumar et al., 2020), while 15.6× for shelling percentage (Luo et al., 2019a) and 14.3× for bacterial wilt resistance (Luo et al., 2019b). The QTL‐seq experiments performed in diploid crop species, such as chickpea (*Cicer arietinum* L.), cucumber (*Cucumis sativus* L.), and pigeonpea (*Cajanus cajan* L. Huth) with small to medium genome size, were conducted based on just 3.7–6.52× average depth (Das et al., 2015; Lu et al., 2014; Singh et al., 2016). Therefore, the results in the present study reconfirm that 9–10× coverage is sufficient for performing QTL‐seq analysis in groundnut.

To understand the superiority of the QTL‐seq approach over genetic mapping, the identified genomic regions for seed weight were compared with previous genetic mapping studies conducted to identify the genomic regions associated with seed weight. In the present study, we identified genomic regions on chromosome B06, B08, and B09 associated with seed weight. Recently, a nested‐association mapping approach identified three and two SNPs on chromosome B06 and B09, respectively, associated with pod weight and seed weight (Gangurde et al., 2020). However, in the present study, we identified a total of 104 SNPs in three genomic regions on B06 and 30 SNPs in the genomic region on chromosome B09. Several studies reported consistent major‐effect QTL for seed weight on chromosome B06 (Zhang et al., 2019; Wang et al., 2018; Huang et al., 2015). Here, we discovered three genomic regions of sizes 8.9 (31.5–40.52 Mb), 1.1 (53.56–54.66 Mb), and 1.64 Mb (81.17–82.81 Mb) on chromosome B06 with a total of 104 SNPs, indicating a plausible role of chromosome B06 in regulation of seed weight. Shelling percentage and seed weight are interdependent traits; genomic regions and candidate genes on chromosome A09 and B02 were identified for shelling percentage using the QTL‐seq approach (Luo et al., 2019b). More importantly, biparental genetic mapping in the same population could identify only one major and consistent QTL on chromosome A09 for shelling percentage (Luo et al., 2017). A recent study using nested‐association mapping for seed and pod weights identified associated genomic regions on chromosomes A05 and B05, A06 and B06, A08 and B08, and B09 (Gangurde et al., 2020), which may be due to contribution from multiple parents. Nevertheless, in the present study, a very narrow genomic region of 930 Kb was identified on chromosome B09 while analyzing reference genome with HS and LS pools. Another genetic mapping study reported major‐effect and consistent QTL on chromosome B07 and B08 for seed weight (Mondal et al., 2015), which matches with the genomic region 2.63 Mb (10.94–13.58 Mb) identified in the present study on chromosome B08. Of the two genetic mapping studies reported so far in groundnut, one study using biparental population identified nine minor effect QTLs for seed and pod size located on the chromosomes A05, A06, A09, B10, B04, A03, B05, and B08 (Chu et al., 2020). Recently, with tetraploid genome assembly, the regions associated with groundnut seed weight were identified on chromosome A07 and B02 (Zhuang et al., 2019).

Keeping in mind the importance of information on candidate genes and significantly associated SNPs for marker development, we targeted associated regions located on chromosomes B06, B08, and B09. The 13 SNPs discovered from these regions affected the function of nine known candidate genes, namely *Ulp protease*, *serine/threonine‐protein kinase*, *basic leucine‐zipper domain*, *methyltransferase*, *EPIDERMAL PATTERNING FACTOR*, *aluminium induced protein with YGL and LRDR*, *MADS‐box transcription factor*, *TraB family protein*, and *tripeptidyl peptidase*. The *Ulp* (ubiquitin like proteases) *proteases* (*Araip.5J71F*) gene identified on chromosome B06 are *SUMO (small ubiquitin‐like modifier) specific cysteine (Cys) proteases* with *SUMO peptidase* activity, which are made by posttranslational modifications and results in dynamic changes to protein function (Augustine and Vierstra, 2018; van den Burg et al., 2010; Gareau and Lima, 2010). It is reported that 40 SUMO proteins showed higher expression of *SUMO1/2* and *SCE1* (*SUMO Conjugating Enzyme 1*) genes during pod development in groundnut. In addition, structural modifications among SUMO conjugates were also observed in response to abiotic stresses (Liu et al., 2019). On chromosome B09, one SNP was identified at position B09_145954466 located in genic region of *EPIDERMAL PATTERNING FACTOR* (EPF) (*Araip.9SE5V*). *TaEPFL1* (*Triticum aestivum* epidermal patterning factor 1) gene was highly expressed in abnormal pistillody stamens as compared with pistil and stamens in *HTS1* mutant of wheat (*Triticum aestivum* L.) (Sun et al., 2019). An isoform of EPF, *BnEPFL6* (*Brassica napus epidermal patterning factor 6*) gene was found essential for the elongation of filaments in rape (*Brassica napus L*.) (Huang et al., 2014). We identified one SNP (B08_10998096) in genic region of serine/threonine protein kinase (*Araip.UL7VH*) on chromosome B08 (B08_10998096), which is known to be involved in the plant growth and development in *Arabidopsis thaliana* (L.) Heynh. (Uhlken et al., 2014). A SNP (B06_81932178) was identified in the intergenic region near to protein kinases (*Araip.9M8Z9*). It is well accepted that receptor‐like protein kinases, such as *HAESA*, are involved in floral organ development (Jinn et al., 2000; Taylor et al., 2016). A receptor‐like protein kinase coding brassinosteroid gene *BRI1* regulates cell growth in *Arabidopsis* (Li and Chory, 1997). *CLAVATA1* genes are receptor kinase coding genes involved in meristem development, cell proliferation, and differentiation (Clark et al., 1997). On chromosome B08, two SNPs (B08_11007755 and B08_11007758) were identified in the genic region of basic leucine zipper domain (bZIP) transcription factor (*Araip.IA4XE*), which are associated with essential processes like seed development, energy balance, and abiotic or biotic stresses resistance (Noman et al., 2017). Another SNP (B09_146312888) on chromosome B09 was identified in genic region of *MADS box‐domain* transcription factors (*Araip.K558L*), which are basic members of regulatory networks underlying multiple developmental pathways in plants, animals, and fungi (Nakashima & Yamaguchi‐Shinozaki, 2013; Janiak et al., 2016; Wils & Kaufmann, 2017; Cho, 2018). In *Arabidopsis*, *MADS‐box* genes are involved in developmental processes such as meristem specification, flowering transition, and seed, root, and flower development (Smaczniak et al., 2012). Three SNPs (B09_145852122, B09_145861466, and B09_145867485) were identified in intergenic region of *homocysteine S‐methyltransferase*, known to be involved in seed development. Reciprocal cross between the wild type and met1‐6 mutant carrying a mutation in *DNA METHYLTRANSFERASE 1* (*MET1*) gene results in hypomethylated maternal genomes cause significantly larger, while paternal genomes cause smaller F_1_ seeds (Xiao et al., 2006). A recessive mutation in another gene that dramatically reduces DNA methylation, *DECREASE IN DNA METHYLATION1*, also causes parent‐of‐origin effects on F_1_ seed size (Table 2). Based on the possible function of the associated candidate genes with seed weight, an epigenetic change in the genome seems to play important role in defining the seed size in groundnut.

**TABLE 2:** Single‐nucleotide polymorphism (SNPs) with ΔSNP index = −1 on chromosome B06, B08, and B09 identified using high‐seed‐weight (HS) and low‐seed‐weight (LS) bulk and ICGV02251 assembly with corresponding genes

**Chromosome**	**Physical position**	**ICGV 02251 base**	**LS base**	**HS base**	**Gene interval for intergenic SNPs**	**Feature of nearest gene**
	bp					
Araip.B06	32,044,422	T	C	T	*Araip.0FI9Y*–*Araip.NVZ99*	MYB transcription factor MYB118
	32,152,814	C	T	C	*Araip.KLU9X*–*Araip.2412K*	Adenylate kinase family protein
	32,349,226	G	A	G	*Araip.2412K*–*Araip.WIZ5S*	WRKY family transcription factor family protein
	32,489,453	C	T	C	*Araip.M1NAC*–*Araip.7L5D1*	Unknown protein
	33,164,677	G	T	G	*Araip.U0213–Araip.TE1P2*	CDPK‐related kinase 3
	33,884,746	A	G	A	*Araip.U9R7I–Araip.K48V4*	Uncharacterized protein
	53,557,434	G	T	G	*Araip.L9809–Araip.V79U1*	WD repeat‐containing protein
	54,290,325	A	T	A	*Araip.116MM–Araip.AEB2X*	Triosephosphate isomerase
	54,401,527	G	T	G	*Araip.AEB2X–Araip.97NYT*	TNP1
	81,630,940	C	G	C	*Araip.8DG38–Araip.7PD4P*	Growth‐regulating factor 4
	81,889,671	A	T	A	*Araip.RVJ8S–Araip.VA5I5*	BTB/POZ domain‐containing protein
	82,026,094	T	C	T	*Araip.9M8Z9–Araip.FJY6W*	Protein kinase superfamily protein
Araip.B08	11,256,389	G	A	G	*Araip.ZL8H3–Araip.AC1PS*	Fe(II)‐dependent oxygenase superfamily protein
	11,815,376	T	G	T	*Araip.DK4JW–Araip.D4EPK*	Seed biotin‐containing protein
	11,840,276	G	A	G	*Araip.D4EPK–Araip.LJQ0L*	Protein YLS7
	12,062,456	G	A	G	*Araip.I0QD1–Araip.FM3FX*	Exocyst complex component sec10
	12,188,146	C	T	C	*Araip.C3D0C–Araip.CMJ6A*	ATP‐binding/protein serine/threonine kinase
	12,787,501	G	A	G	*Araip.GIZ0F–Araip.ATS2M*	Peroxidase superfamily protein
	13,084,370	G	A	G	*Araip.01CTA–Araip.PIP5Q*	Disease resistance protein (TIR‐NBS‐LRR class)
	13,243,168	G	A	G	*Araip.6EA1G–Araip.AH9TM*	Pentatricopeptide repeat (PPR) superfamily protein
	13,403,557	G	C	G	*Araip.EF3LF–Araip.Z2IAU*	Glutamate synthase 1
Araip.B09	145,797,595	C	T	C	*Araip.YWU3B–Araip.M2BYP*	Uncharacterized protein
	145,810,717	A	T	A	*Araip.T82B5–Araip.BY68W*	Vicilin 47 kDa protein
	145,852,122	T	C	T	*Araip.BY68W–Araip.0U7×2*	Homocysteine S‐methyltransferase 3
	145,880,280	G	A	G	*Araip.0Y9HM–Araip.45TAK*	Tesmin/TSO1‐like CXC domain‐containing protein
	145,924,830	C	T	C	*Araip.Q0MMZ–Araip.ZG2VU*	Choline transporter‐like protein 2‐like
	145,959,066	C	T	C	*Araip.9SE5V–Araip.7IG36*	EPIDERMAL PATTERNING FACTOR‐like protein
	145,969,360	T	C	T	*Araip.7IG36–Araip.YK09Y*	Protein TIFY 4B‐like isoform
	145,995,740	G	A	G	*Araip.UP18N–Araip.24AK5*	1,2‐dihydroxy‐3‐keto‐5‐methylthiopentene dioxygenase
	146,102,002	A	G	A	*Araip.I8ABE–Araip.J63ZS*	Argonaute family protein
	146,161,582	C	T	C	*Araip.21M98–Araip.A561Y*	Alpha‐L‐fucosidase 1
	146,430,068	G	A	G	*Araip.G4AXZ–Araip.MI2NR*	Zinc finger CCCH domain‐containing protein
	146,460,214	C	T	C	*Araip.ZB3J2–Araip.B0R6B*	Arginine/serine‐rich splicing factor 35
	146,651,299	G	A	G	*Araip.N2U4Q–Araip.C19HK*	Actin depolymerizing factor 7
	146,728,008	G	A	G	*Araip.L3SN7–Araip.U4GR2*	Tripeptidyl peptidase

**TABLE 3:** Summary of Kompetitive allele specific polymerase chain reaction (KASP) markers developed from significant single‐nucleotide polymorphisms (SNPs) in the genomic regions of important candidate genes for seed weight

**KASP marker**	**Chr**	**Position**	**‘Chico’, low bulk base**	**ICGV02251, high bulk base**	**Gene ID**	**Feature**	**Genic or intergenic**	**Position in tetraploid genome**		
								**Chr**	**Start**	**End**
		bp							bp	
snpAH0031	B06	32,002,355	A	G	*Araip.5J71F*	Ulp1 protease (cysteine‐type peptidase activity)	Genic	Ahy16	37,070,658	37,070,214
snpAH0033	B06	33,002,322	C	T	*Araip.A3T8C–Araip.A5MIH*	ATP binding; nucleoside‐triphosphatase	Intergenic	Ahy16	37,848,691	37,849,457
snpAH0035	B06	54,033,720	T	G	*Araip.K8UVH*	Unknown Protein	Genic	Ahy16	61,321,826	61,322,309
snpAH0037	B06	81,932,178	A	C	*Araip.VA5I5–Araip.9M8Z9*	Protein kinase superfamily protein	Intergenic	Ahy16	94,695,418	94,694,858
snpAH0038	B06	82,423,709	C	T	*Araip.9M8Z9‐Araip.FJY6W*	Protein kinase superfamily protein	Intergenic	Ahy16	94,739,497	94,737,483
snpAH0039	B08	10,998,096	T	A	*Araip.UL7VH*	Serine/threonine‐protein kinase	Genic	Ahy18	12,153,016	12,154,671
snpAH0040	B08	11,007,755	C	T	*Araip.IA4XE*	Basic‐leucine zipper domain	Genic	Ahy18	12,162,664	12,163,477
snpAH0041	B08	11,007,758	T	C	*Araip.IA4XE*	Basic‐leucine zipper domain	Genic	Ahy18	12,162,664	12,163,477
snpAH0042	B08	11,594,032	T	G	*Araip.P0NXK*	Uncharacterized protein	Genic	Ahy18	12,745,717	12,746,277
snpAH0043	B08	11,696,813	C	T	*Araip.WJ0B5*	Unknown Protein	Genic	Ahy18	12,842,397	12,841,709
snpAH0047	B09	145,954,466	T	C	*Araip.9SE5V*	EPIDERMAL PATTERNING FACTOR	Genic	Ahy19	157,520,950	157,520,709
snpAH0048	B09	146,312,888	G	A	*Araip.K558L*	MADS‐box transcription factor	Genic	Ahy19	157,884,580	157,884,310
snpAH0049	B09	146,448,322	C	T	*Araip.MI2NR*	TraB family protein	Genic	Ahy19	158,029,721	158,030,576
snpAH0050	B09	146,719,055	A	T	*Araip.L3SN7*	Tripeptidyl peptidase	Genic	Ahy19	158,283,152	158,282,550

After identifying the candidate genes, we have checked the tissue specificity of these genes based on the expression information available through gene expression atlas for subspp. *fastigiata* (Sinha et al., 2020) and *hypogaea* (Clevenger et al., 2016). Among 182 SNPs, 169 were identified adjacent (intergenic) to 48 candidate genes involved in various physiological processes during plant development. Upon comparing the expression profile of 49 genes in various seed and pod developmental stages using groundnut gene expression atlas (Sinha et al., 2020) (Supplemental Figure S4; Table 2; Supplemental Tables S6 and S7), 21 genes were found to be differentially expressed during embryo, seed, and pod development (Figure 6). Seed biotin containing protein, disease resistance *NBS‐LRR protein*, *vicillin*, *TNP1*, and *WD repeat* containing protein genes were expressed in mature seeds. Whereas transcription factors *WRKY*, *EPIDERMAL PATTERNING FACTOR*, and signal peptide peptidase were highly expressed in flower tissue. *Pentatricopeptide repeats*, *TIFY family proteins*, *Tesmin/TSO1 domains*, *glutamate synthase*, and *protein kinases* are specifically expressed in seeds. Embryo is also an important tissue in the developing seed. *Choline transporters*‐like proteins are specifically expressed in developing embryo tissue. Very few genes were expressed in pod walls with moderate expression values. *Protein kinases*, *ATP sulfurylase*, and *peroxidase superfamily* proteins were found to be expressed in pod walls. In the present study, we identified a sharp peak on chromosome B09 that housed *TIFY family proteins*. The homologues of *BIG SEED* locus belong to *TIFY family proteins* on chromosome B09. BIG SEED 1 (*BS1*) and BIG SEED 2 (*BS2*) loci encodes *TIFY family proteins* reported in barrel clover (*Medicago truncatula* Gaertn.) as negative regulators of seed weight (Ge et al., 2016).

Diagnostic markers accelerate the process of developing improved varieties through facilitating selection using marker‐assisted early generation selection based on single‐seed chipping and rapid generation advancement (Parmar et al., 2021). Deployment of diagnostic markers, including use of KASP markers, for resistance to nematode, leaf rust, and late leaf spot, in addition to high oleic acid content is now been routinely used for improving foliar disease resistance and oil quality using marker‐assisted selection (Chu et al., 2011; Varshney et al., 2014; Janila et al., 2016; Kolekar et al., 2017; Bera et al., 2018; Bera et al., 2019; Deshmukh et al., 2020; Shasidhar et al., 2020; Jadhav et al., 2021). Realizing the importance, we developed and validated four KASP markers (snpAH00031 and snpAH00033 from 8.9‐Mb region and snpAH00037 and snpAH00038 from 1.64‐Mb region of chromosome B06) for seed weight, targeting potential candidate genes to deploy marker‐assisted selection. Therefore, one SNP from each associated region can be used together for selection of large‐seeded plants. More importantly, the snpAH0031 should be given high priority, as it is present in the genic region of *Ulp‐proteases* (SUMO Proteins), which is known for involvement in the regulation of seed size in groundnut. The four KASP markers successfully distinguished between small‐ and large‐seeded groundnut genotypes. These KASP markers also showed heterozygous calls for F_1_ populations derived from the crosses between small‐ and large‐seeded groundnut genotypes, which indicated the plausible use of the developed KASP markers for F_1_ population confirmation and identification of heterozygotes in segregating breeding population during marker‐assisted selection.

## 5 CONCLUSION

The present study demonstrated next‐generation sequencing‐based trait mapping approach as one of the most efficient and fast‐track techniques leading to genomic regions and discovery of putative candidate genes and SNPs associated with 100SW in groundnut. It is reasonable to target the genes from the B genome, such as *Ulp proteases* and *BIG SEED* locus candidate genes, for introgression of bold‐seeded trait in groundnut. However, more evidence is required to functionally validate these genes; plausibly silencing of *BIG SEED* locus using RNAi would be best to validate the function of *BIG SEED* locus. To characterize the impact of candidate gene *Ulp protease* or *SUMO proteins* identified in the present study, overexpression studies as well as use of CRISPR/Cas9 may provide more insights on precise function. More importantly, the validated KASP markers are available through this research to deploy in routine breeding programs for breeding bold‐seeded varieties to meet the preferences of industry and consumers.

## AUTHOR CONTRIBUTIONS

Sunil S. Gangurde: Data curation; Formal analysis; Software; Methodology; Writing – review & editing. Aamir W. Khan: Data curation; Formal analysis; Software. Pasupuleti Janila: Investigation; Methodology; Resources. Murali T. Variath: Resources. Surendra S. Manohar: Resources. Prashant Singam: Data curation; Investigation; Methodology. Annapurna Chitikineni: Investigation; Methodology; Resources. Rajeev K. Varshney: Conceptualization; Funding acquisition; Resources; Writing – review & editing. Manish K. Pandey: Conceptualization; Funding acquisition; Investigation; Methodology; Project administration; Resources; Supervision; Validation; Writing – review & editing.

## CONFLICT OF INTEREST

The authors declare there is no conflict of interest.

## Supporting information


**Supplemental Figure S1**. SNP index plots for 20 chromosomes of low bulk with parent ICGV 02251 a as reference. Red lines indicate the sliding window average of 2 Mb interval with 50 kb increment for SNP index. Unmapped A08 represents the unmapped scaffolds for A‐genome. Unmapped A02 and unmapped B02 represent the unmapped scaffolds of A and B sub‐genomes respectively.
**Supplemental Figure S2**. SNP index plots for 20 chromosomes of high bulk with parent ICGV 02251 as reference. Red lines indicate the sliding window average of 2 Mb interval with 50 kb increment for SNP index. Unmapped A02, A08 and Unmapped B02 represent the unmapped scaffolds for A and B subgenome, respectively.
**Supplemental Figure S3**. The ΔSNP index plot obtained by subtraction of high pool SNP index from low pool SNP index using parent ICGV 02251 as reference. Statistical confidence intervals under the null hypothesis of no QTL are shown (green: P < 0.05; orange: P < 0.01). UnmappedA02, A08 and Unmapped B02 represent the unmapped scaffolds for A and B subgenome, respectively.
**Supplemental Figure S4**. Tissue specific expression of candidate genes identified in genomic regions on chromosomes B06, B08 and B09 discovered using QTL‐seq approach. Gene expression data was retrieved from the gene expression atlas subspp. fastigiata (Sinha et al., 2020) and subspp. hypogaea (Clevenger et al., 2016).

**Supplemental Table S1**. Selection of extreme RILs to construct high seed weight bulk (HS) and low seed weight bulk (LS)
**Supplemental Table S2**. Sequencing and alignment statistics of WGRS data generated for both bulks and parental genotype
**Supplemental Table S3**. Chromosome‐wise SNPs distribution between HS and LS bulk for 100 seed weight
**Supplemental Table S4**. Identification of SNPs with (ΔSNP index = ‐1) using HS and LS bulk and ICGV 02251 assembly
**Supplemental Table S5**. Tissue specific expression of candidate genes identified in QTL‐seq extracted from gene expression atlas fastigiata (Sinha et al., 2020).
Supplemental Table S6. Tissue specific expression of candidate genes identified in QTL‐seq extracted from gene expression atlas subspp. *hypogaea* (Clevenger et al., 2016)
**Supplemental Table S7**. Primer sequences of KASP markers developed for seed weight.
**Supplemental Table S8**. Validation panel of bold and small‐seeded groundnut genotypes (includes 18 Bold seeded genotypes, 5 F1s derived from crosses between small and bold‐seeded genotypes).

## Data Availability

Sequencing data generated in this study have been deposited at National Center for Biotechnology Information (NCBI) Sequence Read Archive (SRA) database with BioProject ID: PRJNA752462.
