# Supplementary material for: Whole‐genome sequencing based discovery of candidate genes and diagnostic markers for seed weight in groundnut
Source: Plant Genome. 2022 Dec 7;16(4):e20265. doi: 10.1002/tpg2.20265 (PMC12807278; doi:10.1002/tpg2.20265)
Supplement: Supplementary file 1 [file TPG2-16-e20265-s002.docx]

**Supplementary Figures**

**Whole genome sequencing-based discovery of candidate genes and diagnostic markers for seed weight in groundnut**

Sunil S. Gangurde^1, 2,3#^, Aamir W. Khan^1,#^, Pasupuleti Janila^1^, Murali T. Variath^1^, Surendra S. Manohar^1^, Prashant Singam^2^, Annapurna Chitikineni^1^, Baozhu Guo^2^, Rajeev K. Varshney^4*^, Manish K. Pandey^1*^

^1^International Crops Research Institute for the Semi-Arid Tropics (ICRISAT), Hyderabad 502324, India

^2^ USDA-ARS, Crop Genetics and Breeding Research Unit, Tifton, GA 31793, USA

^3^Department of Genetics, Osmania University, Hyderabad-500007, India

^4^State Agricultural Biotechnology Centre, Crop Research Innovation Centre, Food Futures Institute, Murdoch University, Murdoch, Western Australia, 6150, Australia

*Corresponding authors: [m.pandey@cgiar.org](mailto:m.pandey@cgiar.org); [r.k.varshney@cgiar.org/rajeev.varshney@murdoch.edu.au](mailto:r.k.varshney@cgiar.org/rajeev.varshney@murdoch.edu.au)

#Authors contributed equally

**Figure S1. SNP-index plots for 20 pseudomolecules of low bulk with parent ICGV 02251 a as reference.** Red lines indicates the sliding window average of 2 Mb interval with 50 kb increment for SNP-index. Unmapped A08 represents the unmapped scaffolds for A genome. Unmapped A02 and unmapped B02 represents the unmapped scaffolds of A and B sub-genomes respectively.

**Figure S2. SNP-index plots for 20 pseudomolecules of high bulk with parent ICGV 02251 as reference.** Red lines indicate the sliding window average of 2 Mb interval with 50 kb increment for SNP-index. Unmapped A02, A08 and Unmapped B02 represent the unmapped scaffolds for A and B subgenome, respectively.

**Figure S3. The (Δ-SNP index) plot obtained by subtraction of high pool SNP-index from low pool SNP-index using parent ICGV 02251 as reference.** Statistical confidence intervals under the null hypothesis of no QTL are shown (green: *P* < 0.05; orange: *P* < 0.01). UnmappedA02, A08 and Unmapped B02 represent the unmapped scaffolds for A and B subgenome, respectively.

**Figure S4.** Tissue specific expression of candidate genes identified in genomic regions discovered using QTL-seq approach on chromosomes B06, B08 and B09. Gene expression data was retrieved from the from gene expression atlas (**Sinha et al. 2020**).
